# Preliminary Techno-Economic Assessment of Animal Cell-Based Meat

**DOI:** 10.3390/foods10010003

**Published:** 2020-12-22

**Authors:** Derrick Risner, Fangzhou Li, Jason S. Fell, Sara A. Pace, Justin B. Siegel, Ilias Tagkopoulos, Edward S. Spang

**Affiliations:** 1 Department of Food Science and Technology, University of California, Davis, CA 95616, USA; drisner@ucdavis.edu (D.R.); sspace@ucdavis.edu (S.A.P.); 2 Department of Computer Science, University of California, Davis, CA 95616, USA; fzli@ucdavis.edu (F.L.); itagkopoulos@ucdavis.edu (I.T.); 3 Genome Center, University of California, Davis, CA 95616, USA; jsfell@ucdavis.edu (J.S.F.); jbsiegel@ucdavis.edu (J.B.S.); 4 Departments of Chemistry, Biochemistry and Molecular Medicine, University of California, Davis, CA 95616, USA; 5 Innovation Institute for Food and Health, University of California, Davis, CA 95616, USA; 6 USDA, AI Institute for Next Generation Food Systems (AIFS), University of California, Davis, CA 95616, USA

**Keywords:** cultured meat, cell-based meat, techno-economic assessment, bioreactor, process engineering, bioengineering, biomanufacturing

## Abstract

Interest in animal cell-based meat (ACBM) or laboratory-grown meat has been increasing; however, the economic viability of these potential products has not been thoroughly vetted. Recent studies suggest monoclonal antibody production technology can be adapted for the industrialization of ACBM production. This study provides a scenario-based assessment of the projected cost per kilogram of ACBM produced in the United States based on cellular metabolic requirements and process/chemical engineering conventions. A sensitivity analysis of the model identified the nine most influential cost factors for ACBM production out of 67 initial parameters. The results indicate that technological performance will need to approach technical limits for ACBM to achieve profitably as a commodity. However, the model also suggests that low-volume high-value specialty products could be viable based on current technology.

## 1. Introduction

Global population growth and economic development are expected to double the demand for meat products by 2050 [1]. Meanwhile, the United Nations Food and Agriculture Organization (FAO) estimates that beef and dairy cattle may be responsible for up to 5.0 gigatonnes of CO_2_-equivalent emissions, or 9% of total greenhouse gas (GHG) emissions [2,3]. These reported emissions are considered generalizations, and a nuanced examination of an individual production system must occur to quantify CO_2_-equivalent emissions for each system [4]. Concerns over global warming, animal welfare, and human health have prompted interest in the development of “meat alternatives” which have the organoleptic qualities of meat, but whose origin is not from a slaughtered animal [5,6,7,8]. The environmental costs of ACBM production are still being determined and debated [9,10,11,12,13,14], however significant economic interests in ACBM products continue to grow. Analyst reports are bullish on growth in the meat alternatives sector and have predicted a significant displacement of conventional ground beef, with some reports predicting a 60–70% decrease over the next 10–20 years [7,8]. The predicted shift to meat alternatives would represent a disruption of a highly valuable market. In 2018, the United States processed 12.1 million tonnes of beef, including 8.5 million tonnes of retail cuts valued at USD 106 billion [15].

Plant- and fungal-based meat alternatives are already widely available, but producers and consumers are looking to animal cell-based meat (ACBM) as the next frontier for meat alternatives. While ACBM has yet to be scaled commercially, it is currently perceived as a core component of this “2nd domestication of plants and animals” [7]. In fact, ACBM companies have received significant early stage investments in excess of USD 230 million [16,17]. This level of economic investment suggests the need for a rigorous assessment of the pathway to profitability for the sector.

**Figure 1:**
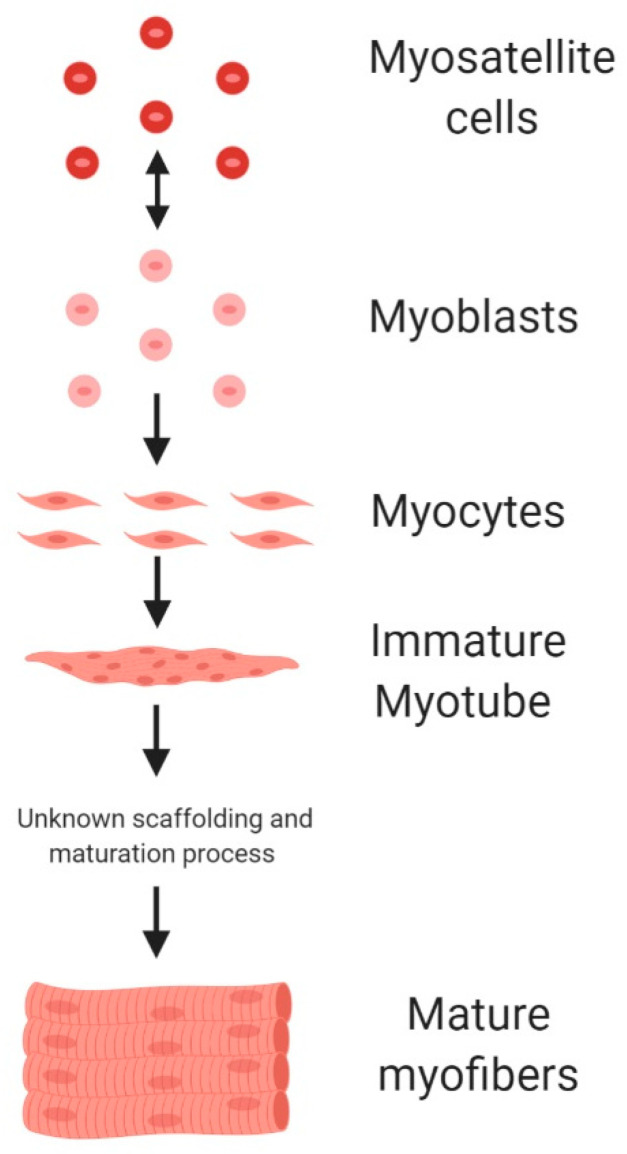
Myogenesis sequence for animal cell-based meat (ACBM) production (figure produced using BioRender).

Proposed ACBM production systems suggest existing pharmaceutical technologies could be employed for mass ACBM production [6,18]. Industrial-scale bioreactors would be used to proliferate myoblasts or myosatellite cells which were harvested from animals [6,18]. These cells then undergo a differentiation and maturation process (i.e., myogenesis) supported by a scaffolding process to form the final ACBM meat product (Figure 1) [19,20,21]. ACBM first became a reality in 2013 with an initial public demonstration of a 140 g “hamburger” that cost over USD 270,000 to produce [22]. The high cost of production remains a significant challenge for the ACBM industry. A number of technological hurdles to lower production cost have been identified but not extensively quantified (e.g., cell senescence, high cost of growth factors, time and nutrients required for cell growth/differentiation/maturation, and scalable scaffolding processes) [20,23,24].

**Figure 2:**
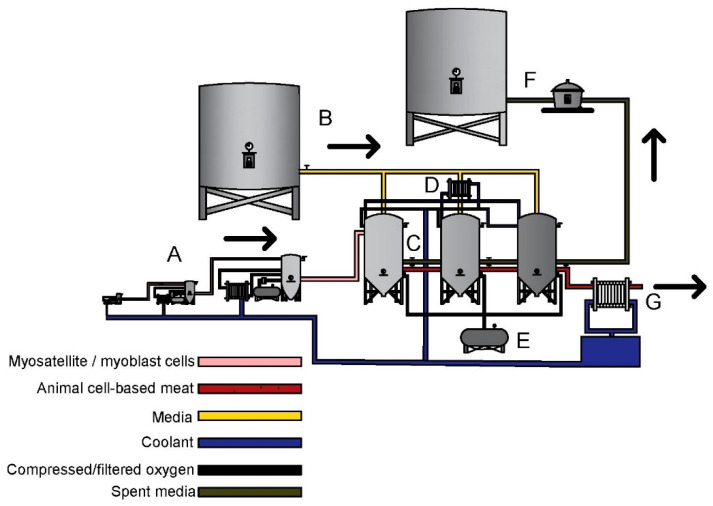
Potential industrialized ACBM production system.

**Figure 3:**
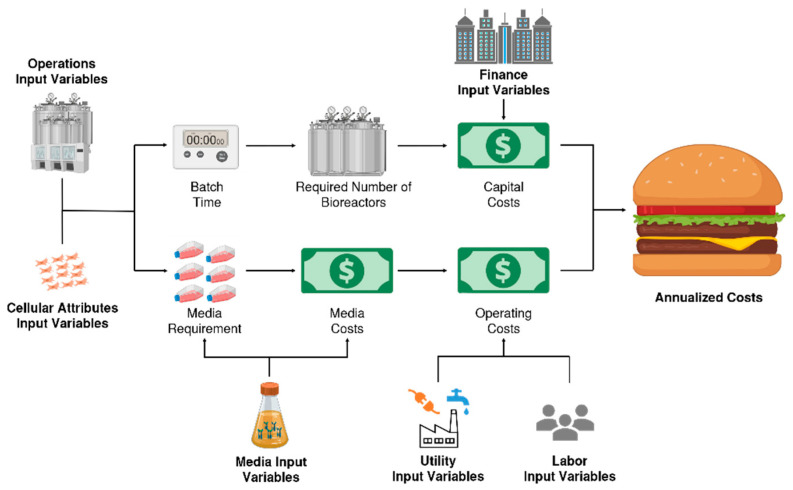
ACBM simplified economic model flow diagram.

**Figure A1:**
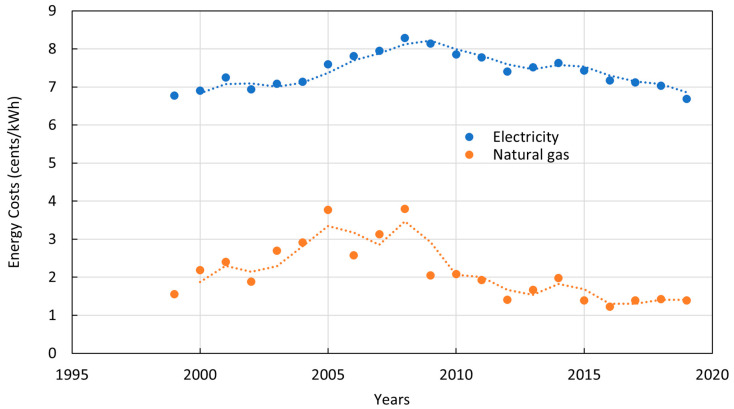
Costs comparison of the average United States industrial electricity and natural gas (USD kWh^−1^) 1999–2019.

**Figure A2:**
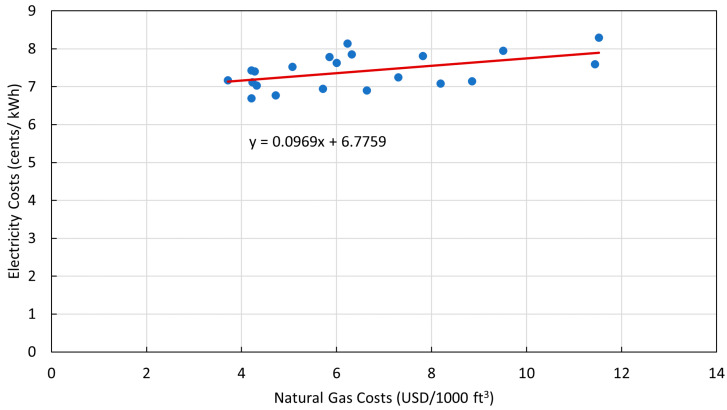
Linear relationship between electricity and natural gas cost.

Figure 2 illustrates a potential ACBM production system similar to monoclonal antibody production for bovine myoblasts/MSC expansion [6,25]. We limit our analysis here to the core bioreactor system (section “C” in Figure 2) since industrial-scale scaffolding and maturation systems have not been defined in detail by ACBM producers. Thus, the model presented is a simplified and reduced model whose reported cost should be considered as minimum costs (Figure 3, Equations (A1)–(A47), and Figure A1 and Figure A2). 

This system represents a potential ACBM production process without pumping system shown: A. The bioreactor seed train system 20 L, 200 L and 2000 L; B. Media storage system; C. Series of 20,000 L continuous stir bioreactor system with unknown scaffolding processing occurring in bioreactor system; D. Bioreactor temperature control system; E. Oxygen supply system; F. Spent media processing system; G. ACBM cooling system. Capital expenditures only account for C, and therefore a minimum estimate of capital costs. Figure produced using AutoCad.

Figure 3 illustrates the ACBM simplified economic model flow diagram. The individual input variables have been grouped into categories (Operations, Cellular attributes, Finance, Media, Utility and Labor) and can be viewed individually in Table A1, Table A2, Table A3, Table A4, Table A5, Table A6 and Table A7. Figure produced using BioRender.

Cultured bovine myoblasts using microcarriers (Cytodex^®^ 1 or Synthemax^®^ MC) behave similarly to human mesenchymal stem cells (HMSC) [26]. HMSC bioprocessing is highly complex given the heterogeneity of the HMSC cultures, sensitivity of HMSC to environmental changes, spontaneous differentiation, and the necessary disassociation of cell aggregates for harvest [27,28]. Meanwhile, the high risk of batch contamination has led many therapeutic stem cell manufacturers to shift to single-use bioreactor systems [28]. However, this study makes the optimistic assumption that advances in MSC/myoblast science will enable the production of MSC/myoblast using large, non-disposable, and semi-continuous bioreactor systems, and that operational issues related to bioreactor sanitation and fill rates are negligible.

## 2. Materials and Methods

To determine the economic viability of animal cell-based meat (ACBM), we developed a model using standard process and chemical engineering methods. The model system is a semi-continuous-batch production system operating at capacity year around and does not account for fill times, sanitation between batches or any operational downtime. Appendix A identifies some of our model’s limitations and a sensitivity analysis was also conducted to further understand the influence of each model variable (Appendix B). All Equations and variables are available in the equation and variable lists (Appendix C) as well as in the python code associated with our model. Table A9 (Appendix D) provides a list of equipment that would likely be necessary for industrial ACBM production. The costs were broadly broken down into annual operating costs and capital expenditures then annualized.

### 2.1. Capital Expenditures of an ACBM Plant

We accounted for the volume each myoblast/myosatellite cell (MSC) occupies with the operating constraint that the total cell volume cannot exceed bioreactor operating capacity for each batch. Cell volumes are variable, so a reported volume estimate of 5 × 10^−15^ m^3^ cell^−1^ was used [18]. Eukaryotic muscle cell density is approximately 1060 kg m^−3^ and was used to estimate mass of ACBM per batch [29]. The actual density of ACBM may be lower due to incorporation of bovine adipose cells or other sources of fat. A decrease or increase in batch time influences economic viability of ACBM production. The batch time is the sum of the cell growth phase and maturation time (Equation (A1)). The cell concentration is considered a variable that can change with technological innovation. Using a given cell concentration, the mass of each batch of ACBM was determined using Equations (A2)–(A4). The batch time was then used to calculate the annual ACBM batches per bioreactor and the number of bioreactors required to achieve the desired annual ACBM production mass (Equations (A5) and (A6)).

Cost estimates of food-grade bioreactors were calculate using a method which accounts for equipment scaling, installation, and inflation (Equations (A7) and (A8)) [30]. This method applies a set unit cost of USD 50,000 m^−3^ for a food grade bioreactor and a common scaling factor of 0.6 [30]. To account for inflation and changes in cost over time the Chemical Engineering Plant Cost Index (CEPCI) values for heat exchangers and tanks were used to determine an adjusted value factor [31,32]. Adjusted value factor of 1.29 was determined dividing the recent CEPCI values with the values from when the set unit cost was referenced. The Lang factor is used to estimate cost associated with installation and piping. This factor can range from 1.35–2.75 for traditional food production operations and to up to 4.80 for fluids processing [33]. A Lang factor was estimated to be 2 for all scenarios. For new plant cost the Lang factor value should be increased by 1 [33]. This estimated the minimum capital expenditures for the required number of bioreactors which are necessary to meet the desired ACBM production mass. This method does not account for any other equipment which would likely be necessary for ACBM production (Table A9) besides the primary bioreactor systems.

### 2.2. Operating Costs of an ACBM Plant

The potential manufacturing cost of an ACBM plant can be broken into three categories: fixed manufacturing costs, variable capital costs and indirect (overhead) costs. All fixed manufacturing costs were estimated as a percentage of the fixed equipment costs except loan and equity interest (Equation (A9)) [33]. These costs include equipment maintenance, insurance, taxes and royalties costs [33]. Indirect costs which are cost not related to amount of product processed, such as sales expenses and local taxes and are not accounted for in our model since these costs are outside of plant operation expenses and will vary company to company. Our model provides an estimate of several variable capital costs related to downstream ACBM production. Costs associated with general meat production such as packaging material and facility lighting are not included. The variable costs estimated in our model include ingredients, raw materials, utilities, and labor costs. Equation (A10) accounts for all the operating costs associated with the model we have provided.

#### 2.2.1. Ingredients and Raw Materials

A key material for animal serum-free ACBM production is the specialized media required for myoblasts/MSCs growth. We assume bovine myoblast/MSCs have been harvested from cattle and preserved in a manner which will allow for propagation in animal serum-free media. Our model examines the use of Essential 8, an animal free growth medium which contains over 50 ingredients including ascorbic acid 2-phosphate, sodium bicarbonate, sodium selenite, insulin, transferrin, fibroblast growth factor-2 (FGF-2), and transforming growth factor beta (TGF-β) [18]. A report from the Good Food Institute provides an excellent breakdown of the individual components of Essential 8 media and the 2019 pricing of each media component [18]. Cell glucose consumption rates can vary based upon several factors including glucose concentration present in the growth medium and the metabolic pathways being utilized by the cell [34,35]. Glucose consumption rates have been reported to be between 2 to 20 nmol^−1^ million cell^−1^ min^−1^ in human stem cells [35]. While there can be many limiting factors in a complex medium system; glucose consumption and the total number of cells in the bioreactor were used to estimate the media requirements and expense per batch. The starting glucose concentration is reported to be 1.78 × 10^−2^ mol L^−1^ [18]. Only media used in the main bioreactor was accounted for. An oxygen supply is also critical for aerobic cell culture and is also considered an operating expense for ACBM production. Equation (A11) was used to determine total amount of myoblasts/MSCs in the bioreactor at a given time. During the growth phase, the glucose consumption rate changes as time changes and this was accounted for using Equation (A12). The total glucose required for the growth phase was determined by Equation (A13). The total glucose required per batch was determined by adding the total glucose used in the maturation and growth phase (Equations (A14) and (A15)).

The media requirement was then determined by examining the total amount of glucose in the Essential 8 media. To understand the volume requirement per batch, a charge was deemed the equivalent to the working volume of the bioreactor. This assumption was done to account for any innovations related to vascularization and does not account for the volume of the cells. The total media volume required per batch/year and total annual media costs were determined by Equations (A16)–(A19).

An oxygen supply is critical for aerobic cell cultures and is also considered an operating expense for ACBM production. The oxygen levels in the bioreactor were assumed to be kept in a steady state concentration of 2% for optimal cell growth [27,36]. This is expressed by Equation (A20) [37]. The initial oxygen needed for the bioreactor system was determined by Equation (A21). The annual oxygen requirement was determined in the same manner as the media requirement and is calculated using Equations (A11) and (A22)–(A27).

#### 2.2.2. Utility Related Expenses

Our model accounts for some bioreactor operating expenses. These should be viewed as theoretical minimum estimates based upon conventional thermodynamic equations. The energy requirements for heating the media, cooling the bioreactors, and cooling of the ACBM mass leaving the bioreactor systems were estimated. The water/media was assumed to enter the facility at approximately 20 °C. The media is also assumed to have an isochoric specific heat of approximately water. The density of the media was assumed to be 1 kg L^−1^ and would be heated to 37 °C. The minimum energy required to heat the media was calculated using Equation (A28). The metabolic consumption of glucose and oxygen produces heat which must be removed from the system. Approximately 470 kJ of heat is released per mol of O_2_ consumed during glucose combustion (Equation (A29)) and this value was used to approximate cellular heat generation [37]. The minimum energy required to be removed from the system to ensure cell health was calculated using Equation (A30). The ACBM mass leaving the bioreactor must be cooled from 37 °C–4 °C to ensure food safety standards are maintained [38]. The specific heat of ACBM is assumed to be the same as beef which is 2.24 kJ kg^−1^ °C^−1^ [39]. An estimation of energy used during the cooling process (Equation (A31)) was made based on the efficiency of the heat exchanger system.

Energy costs can be variable depending upon the location, time of day and amount used. A yearly national grid average for industrial electricity and natural gas prices was obtained from the United States Energy Information Administration (EIA) from 1999–2019 [40]. One thousand cubic feet of natural gas contains approximately 303.6 kWh of potential energy and the cost per kWh was determined using this value [41]. The average costs were normalized to January 2019 prices using the CPI inflation calculator (Table A10 and Table A11) [42]. To estimate the energy/electricity cost a comparison of the industrial price of natural gas and electricity was made from 1999–2019 (Figure A1). Equation (A32) was derived from a linear relationship of the cost of electricity and natural gas (Figure A2). Equation (A32) was then used to estimate energy/electricity costs from a public supplier. Natural gas was chosen since it is the most used source of energy for electricity production in the United States in 2019 [43]. The costs of energy/electricity produced via an onsite boiler–turbine system was estimated by Equation (A33). A steam pressure of 42.5 bar is assumed because it is used as a reference pressure for cost of steam production and is adequate for steam turbine electricity production [44,45]. Solar generation of electricity was considered as well and was estimated to have a negligible operating cost for the facility. The equipment costs for solar are not accounted for since this is a facility dependent item. Equation (A34) estimates the minimum cost of energy at an ACBM production facility.

Our model assumes media will be produced onsite given the scale of the operation. All water used for media production is considered process water; however, it should be noted that deionized water could be required due to the operational sensitivity of myoblasts/MSCs. Compressed air is a common utility in food production facilities; however, it is not estimated in this analysis due to being a site-specific consideration. Cost of sterile filtration of the water for media production is not accounted for. The spent media is considered wastewater and must be treated to comply with environmental regulations [46]. The wastewater is assumed to be treated by a filtration and biological oxidation step. Cost estimates have been made for process water and wastewater treatment and these estimates have been adjusted to January 2019 values to account for inflation (Table A12) [42,44]. It should be noted that this does not account for water used for sanitation or for losses during the production process. Equation (A35) is used to estimate the annual process and wastewater costs.

#### 2.2.3. Labor Related Expenses

Our scenarios assume that the ACBM production facility is operating 24 h/day and year around. It is assumed the facility is fully staffed and no overtime is required. Each shift is assumed to be an 8-h shift. The facility is also assumed to be in the United States in an area of standard income. The required production operators (required manpower) for the ACBM production facility per shift is estimated by amount and type of processing equipment in the facility (Table A9) [30,44]. This processing equipment could include centrifugal pumps, plate filters, media holding vessels, heat exchangers, bioreactor seed train, positive displacement pumps and bioreactors. In the four scenarios, this equipment was deemed site specific and only the main bioreactors were accounted for. The labor cost were determined using the mean hourly rate, USD 13.68 (USD h^−1^) for a meat packer [47]. The manpower requirement was one laborer per full-scale bioreactor and then the labor costs were estimated using a factorial method with a labor cost correction factor (Equations (A36)–(A38)) [44].

#### 2.2.4. Finance Related Expenses

Our model accounts for the expenses related to equity recovery and debt using a standard finance calculation (Equations (A39)–(A46)) [48]. For all scenarios, the input variables were kept constant. Equations (A39)–(A46) convert the capital expenditures to an annual cost which is used to calculate the total annual minimum costs in conjunction with the annual operating costs (Equation (A47)).

#### 2.2.5. Sensitivity Analysis

We performed a sensitivity analysis of the ACBM price model using 6 algorithms that use different approach to variance and rate of change to assess sensitivity: the Derivative-based Global Sensitivity Measure (DGSM), Delta Moment-Independent Measure (DMIM), Morris Method (MM), Sobol Sensitivity Analysis (SSA), Fourier Amplitude Sensitivity Test (FAST), and the Random Balance Designs Fourier Amplitude Sensitivity Test (RBD-FAST). We used the SALib Python package for this work [49]. Additional information regarding sensitivity analysis algorithms can be found in the Appendix B.

## 3. Results

Using cellular biology and chemical/process engineering conventions, we identified sixty-seven key variables that influence capital or/and annual operating costs (Table A1, Table A2, Table A3, Table A4, Table A5, Table A6 and Table A7). The capital cost of a single 20 m^3^ food-grade bioreactor was estimated to be USD 778,000 [30]. We limit bioreactor size to 20 m^3^ given the sensitivity of animal cells to elevated hydrostatic pressures as compared to fungal/bacterial cells which can be viable in >500 m^3^ scale bioreactors [50]. The annual operating expenses include fixed manufacturing costs, media, oxygen, energy, process water, and wastewater treatment costs.

**Figure 4:**
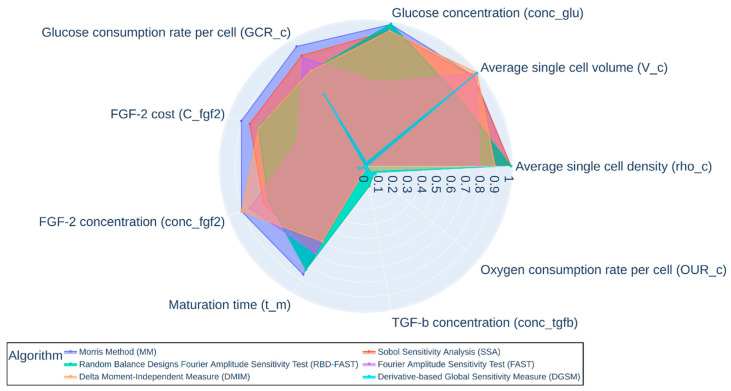
ACBM sensitivity analysis of key model variables.

To understand the impact of each model variable on the estimated capital and annual operating expenses, we performed a robust sensitivity analysis (Figure 4 and Table A8). We applied six global sensitivity analysis algorithms (Derivative-based Global Sensitivity Measure, Delta Moment-Independent Measure, Morris Method, Sobol Sensitivity Analysis, Fourier Amplitude Sensitivity Analysis, Random Balance Designs-Fourier Amplitude Sensitivity Test) to identify the top nine factors that most influenced capital and annual operating expenses by consolidating the top 5 parameters across all six algorithms. These nine factors were then clustered into technological components (including maturation time, fibroblast growth factor 2 (FGF-2) concentration and costs, glucose concentration, glucose consumption rates, oxygen consumption rate and transforming growth factor beta (TGF-β)) and cell-based components (e.g., average cell volume and density).

ACBM sensitivity analysis of key model variables. Each algorithm independently examined 67 parameters for sensitivity. The five parameters exhibiting the most sensitivity were selected from each algorithm. This resulted in nine unique parameters visualized in the figure. The sensitivity measurements of the algorithms were scaled from 0–1 using minimum-maximum normalization except DGSM. The measurement of DGSM was first scaled by taking its sixteenth root and then normalized from 0 to 1 by minimum-maximum. Abbreviations for each of the nine unique parameters are provided for reference to the input variables in Data S1. Figured produced using Python.

The results from the sensitivity analysis then informed the specification of four technology development scenarios (Table 1, Table A1, Table A2, Table A3, Table A4, Table A5, Table A6 and Table A7). Scenario 1 represents a baseline scenario a based on existing ACBM production, including 2019 cost estimates for animal serum-free media and growth factors [18]. Scenario 4 was designed as a bookend scenario, where nearly all technical challenges are resolved, including reduced growth factor costs, increased MSC/myoblast tolerance to glucose concentrations, decreased MSC/myoblast doubling and maturation time, and reduced basal media costs [6,18,20,23]. Scenario 2 represents a mid-point scenario between Scenarios 1 and 4, and Scenario 3 adapts Scenario 2 by eliminating FGF-2 growth factor costs. To incorporate economic scalability, we also examined the capital and annual operating expenditures (Table 2) to produce enough ACBM to replace 1% of the United States beef market (121,000,000 kg) [15].

Scenario Description: Scenario 1 represents a baseline scenario which utilizes a 2019 baseline cost estimate of Essential 8 media from a Good Food Institute report [12]. Scenario 4 is a scenario where nearly all technical challenges are resolved. Scenario 2 represents a mid-point scenario between Scenarios 1 and 4, and Scenario 3 is identical to Scenario 2 except FGF-2 growth factor costs are eliminated.

The results of our calculations indicate that ACBM production will only approach economic viability as a commodity when the significant technical challenges are overcome as outlined in Scenario 4 (Table 1 and Table 2). In Scenario 1, the cost per kilogram remains exceedingly expensive at approximately USD 400,000. Scenarios 2 and 3 illustrates the significant impact of reducing the cost of FGF-2, which reduces the operating cost of ACBM by an order of magnitude from Scenario 1. Only in Scenario 4 does ACBM approach commodity level prices at approximately USD 2 per kg.

The cost of the bioreactor was the main driver of capital costs in the model. To displace the demand for beef in the U.S. by 1%, the scenarios ranged from requiring the deployment of 5205–50 bioreactors (20 m^3^) at a total capital cost of USD 4 billion to USD 37 million. The capital expenditures in scenario 3 remain the same as scenario 2 since eliminating the growth factor cost has no impact on the capital expenditures. Finally, it is important to reiterate that these costs are based on estimates for standard food-grade bioreactors and that more sophisticated bioreactors (i.e., single-use or novel perfusion systems) may substantially increase capital costs.

While capital expenditures are significant, the operating expenses (largely based upon cellular metabolism and media consumption) represent a substantial hurdle for the large-scale production of ACBM. Achieving the outcomes presented in Scenario 4 would require significant technological advancements on multiple fronts as specified by the model, where media costs are reduced from 376.80 USD /L to 0.24 USD /L, glucose/media consumption is reduced by an order of magnitude, and cell growth and maturation times are heavily decreased from 24 h to 8 h and 240 h to 24 h, respectively.

## 4. Discussion

The results of the scenario analysis clearly highlight and quantify the technological and economic challenges for ACBM to reach commercial viability. We suggest the following three areas of focus to reach techno-economic feasibility, which we will discuss further: cell selection or engineering to lower the media consumption rate; reducing or eliminating the cost of growth factors; and scaling up of perfusion bioreactors.

The analysis identified cell metabolism as a key limiting factor for the economic viability of ACBM, so understanding and potentially manipulating cellular metabolism represents a key area of innovation for driving down operating costs. The glucose consumption rate of cultured cells establishes the media requirements in our model, which is by far the largest operating expense for ACBM production. Scenario 1 was based upon reported human embryonic stem cells’ glucose uptake rate. These cells were likely exhibiting a Warburg metabolism (aerobic glycolysis) based upon their lactate production rates [51,52]. This metabolic mode is common during cell proliferation; however, it is energetically less efficient than oxidative phosphorylation (i.e., production of 2 ATP vs. a theoretical 38 ATP per glucose molecule) [52]. Engineering and/or screening for cell lines which shift rapidly from a Warburg metabolism to a more glucose-efficient metabolism represents an opportunity to reduce the media consumption rate in line with Scenario 4.

In healthy cells, glucose uptake is stimulated by growth factors such as insulin, FGF, or/and TGF [51,53]. Our model highlights that growth factors are a major contributor to ACBM production expenses, with FGF-2 being particularly impactful. Thus, eliminating the need for FGF-2 would significantly reduce costs. One potential pathway for this solution would be to leverage the ability of cancer cells to increase glucose uptake rates and exhibit cell proliferation without the presence of growth factors [51]. Thus, cell lines could be engineered or identified to express oncogenes related to these traits. However, utilizing cultured cells that behave similar to cancer cells would likely be very challenging from both a regulatory perspective as well as for consumer acceptance. It should also be noted that Essential 8 media is not generally recognized as safe (GRAS) for human consumption and ensuring cell culture media is composed of ingredients which are GRAS will be an additional regulatory/technical challenge.

Our model indicates that cellular metabolic requirements will require multiple changes of media per batch and higher cell concentrations [54,55]. The use of perfusion bioreactors could deliver these capabilities for ACBM production [56]. Concentrations of 2.0 × 10^8^ cells/mL have been reported for Chinese hamster ovary (CHO) cells in a lab-scale, disposable perfusion bioreactor system [56]. However, this is a profoundly different technology to the large-scale, continuously stirred bioreactors we assume in our model. To the authors’ knowledge, a perfusion bioreactor system with a 20 m^3^ working capacity is not currently in existence for myoblasts/MSC propagation.

ACBM has been presented as a potentially disruptive technology that can transform the global meat sector. However, our techno-economic analysis of this alternative meat production pathway suggests that the profitable mass production of products composed entirely of ACBM remains a significant challenge. Our model indicates that several technical challenges must be overcome before industrial scale-up is likely to be profitable. Media consumption rates must be measured and optimized at the cellular level and the costs of growth factors must be significantly reduced or eliminated altogether.

While these factors indicate that ACBM may not be economically viable as a commodity for some time, it does not preclude the potential to enter the marketplace sooner as a minor ingredient which lends desirable organoleptic qualities to an otherwise plant-based product. Alternatively, there may be opportunity for viable competition in the specialty foods markets, where ACBM costs compare more favorably to such items as almas beluga caviar (USD 10,000/kg), Atlantic bluefin tuna (USD 6500/kg), and foie gras (USD 1232/kg) [57].

## 5. Conclusions

Our model has highlighted some of the significant economic challenges which impede the techno-economic viability of ACBM, but it is not comprehensive. Given the uncertainty of ACBM production, our model and scenario analysis should be considered a starting point for those interested in the scalability of ACBM. Our scenario analysis is based upon the production of ACBM in the United States which influences factors such as energy and labor costs. The energy and labor costs were minor contributors to our limited model’s operating expenses; however, these costs will likely increase on a fully scaled system. To enable further, and customizable, exploration of how advances in technology might inform ACBM production costs, we have developed an open-source, web-based version of our model that is publicly available at https://acbmcostcalculator.ucdavis.edu.

## Figures and Tables

**Table 1:** Model scenario settings.

Scenario	Achievable Cell Concentration (Cells/Ml)	FGF-2 ^1^ Conc. (G/L)	FGF-2 Cost (USD/G)	Glucose Conc. in Basal Media (Mol/L)	Glucose Consumption Rate Per Cell (Mol/H Cell)	Hours Per Doubling (H)	Maturation Time (H)
1	1.00 × 10^7^	1.00 × 10^−4^	2.05 × 10^6^	1.78 × 10^−2^	4.13 × 10^−13^	24.0	240
2	9.5 × 10^7^	5.00 × 10^−5^	1.00 × 10^6^	2.67 × 10^−2^	2.07 × 10^−13^	16	156
3	9.5 × 10^7^	5.00 × 10^−5^	0	2.67 × 10^−2^	2.07 × 10^−13^	16	156
4	2.00 × 10^8^	0	0	3.56 × 10^−2^	4.13 × 10^−14^	8	24

^1^ Fibroblast growth factor 2 (FBF-2).

**Table 2:** Annualized expenditures with quantified drivers of capital and operating expenditures.

Scenario	Total Required Bioreactors	Volume of Media Needed for Annual Production (L)	Minimum Price of ACBM ^1^ To Meet Annual Capital and Operating Expenses (USD/Kg)
1	5205	1.40 × 10^11^	4.37 × 10^5^
2	360	3.06 × 10^10^	5.72 × 10^4^
3	360	3.06 × 10^10^	4.46 × 10^4^
4	50	8.56 × 10^8^	1.95

^1^ Animal cell-based meat (ACBM).

## Supplementary Materials

Data S1: Techno-economic analysis and sensitivity analysis python code for ACBM https://github.com/IBPA/IBPA-Collection-of-Reproducible-Code-and-Results/tree/master/2020_Artificial_Meat. Data S2: Techno-economic analysis web-based program for ACBM https://acbmcostcalculator.ucdavis.edu.

## Appendix A. Model Limitations

In human pluripotent stem cells, as the cells exit pluripotency and enter the initial differentiation phase a metabolic shift to mitochondrial OXP occurs [58,59]. A similar shift occurs as myoblasts fuse differentiate into myotubes [60]. As myoblasts differentiate into myotubes it has been reported that the metabolic rate is maintained despite a greater reliance on OXP pathway for ATP production [60,61]. However, it is not known if this metabolic rate will be maintained during the undefined scaffolding and maturation process. During this undefined scaffolding and maturation process, the myotubes diameter could potentially increase 20-fold [20,21,62]. Our model assumes glucose and oxygen uptake rate are maintained during this process; however, these values could change to meet the metabolic needs of the maturing myotubes. Once the myotubes mature, they rely upon OXP to meet their metabolic needs and this shift may require an adjustment to operation factors such as an increased or decreased media or oxygen supply.

Our model did not account for amino acid uptake rates due to glucose being the most consumed nutrient in cell culture, however amino acid (AA) metabolism should be a consideration for commercial scale up. An example of the importance of this consideration is that stem cell amino acid metabolism can vary species to species [63,64]. Bovine and mouse embryonic stem cells are sensitive to extrinsic deprivation of threonine, whereas human embryonic stem cells are not sensitive extrinsic deprivation of threonine, but require increased levels of methionine [64,65,66]. This extrinsic threonine requirement does not apply to other mouse or bovine cells which are proliferating [63]. This illustrates how these requirements can vary by species and by cell type.

Glutamine is utilized as both a nitrogen donor and energy substrate in proliferating myosatellite/myoblast cells [67,68]. Glutamine is the second most consumed nutrient in animal cell cultures and contributes to nucleic acid, protein and lipid production [69]. Glutamine concentration has been show to influence the myoblasts proliferation rate with 300 µM being reported as the optimal conditions for human myoblasts proliferation [68]. This indicates that amino acid levels in the media could potentially influence operating costs via increased or decreased doubling times. This would likely be cell line dependent and should again be a consideration for companies wishing to develop multiple products from different cell lines.

The volume of animal cells also plays an important factor in our modeling which accounts for the volume of each cell. Animal myoblasts cells volume are orders of magnitude larger than common prokaryotic or single cell fungi [70]. This places hard constraints on the number of cells a single bioreactor can produce per batch, i.e., bioreactor with a working volume of 20 m^3^ can only produce the number of cells whose total volume is 20 m^3^. This does not account for repulsive forces or for the media within bioreactor. While this was done to account for any innovations in vascularization it makes the model less conservative and should be a consideration for any company considering scale up. It also does not account for cellular volume increases during the unknown scaffolding and maturation phase. The diameter of the myotube can increase up to 20 times its original size as contractile protein is formed [20,21,62]. This increase in size of the cells during maturation could make the bioreactor more efficient, however it was not included in our model due to the unspecified nature of the commercial process.

Figure 2 represents a potential upstream production system for ACBM, however the capital expenditures that were estimated by our model only estimate the cost of a series of 20,000 L continuous stirred bioreactors designated by letter A. We did not adjust the maximum bioreactor operating capacity of the bioreactors in any scenario due to fragility of animal cells which lack a cell wall and cannot withstand the hydrostatic pressures that yeast or prokaryotic organisms can [50]. Innovations in bioreactor design could potentially increase the maximum working capacity. An increase in bioreactor working capacity would potentially lower capital expenses and annual operating costs. However, this would initially increase the base cost (USD 50,000/m^3^) of the bioreactor measured in our model. In a more detailed analysis, as the metrics we have outlined are achieved, interest rate and learning curve equations could be applied to estimate capital and operating expenses in finer granularity. We also assume that the unknown scaffolding and maturation process could be accomplished within the bioreactors. If a separate bioreactor or maturation vessel is needed this would also increase capital expenditures. We did not account for the other equipment since this will be a site-specific variable. The Lang factor is used to estimate actual cost of equipment by accounting for installation related expense. A Lang factor of 2 was chosen for all scenarios to represent a food/bioprocessing facility that could be easily configured to accommodate ACBM production. However, a Lang factor of 2 is considered to be low by general conventions for a brand new facility or novel technology; a Lang factor of 3 to 5 would be more appropriate [30]. We anticipated that once the ACBM is cooled it will be processed in a manner similar to other ground meat products. We also did not account for any additional ingredients being added to the product. Cellular propagation technology could potentially be applied for myoblasts/MSC propagation. Cytodex^®^ 1 microcarriers have been employed for bovine myoblasts proliferation and achieved a cell concentration of approximately 9 × 10^6^ cells/mL [26]. Our model does not account for this technology or any additional propagation technology which may increase capital or operating expenses. It has also been reported that bovine muscle satellite cells have been cultured with hemoglobin and myoglobin [71]. Costs associated with additional ingredients or media supplementation have not been accounted for and could substantially increase the annual operating expenses.

## Appendix B. Additional Sensitivity Analysis Information

All sensitivity analysis calculations were conducted using the SALib Python package [49]. Regarding sampling techniques and parameters, Delta Moment-Independent Measure [72,73] and Random Balance Designs Fourier Amplitude Sensitivity Test [74,75,76] used 1000 samples generated using Latin hypercube sampling [77], where Random Balance Designs Fourier Amplitude Sensitivity Test used the inference number of 10. Sobol Sensitivity Analysis used 1000 samples generated using Saltelli sampling [78,79,80]. Morris Method was sampled with 1000 trajectories and 4 grid levels [81]. Fourier Amplitude Sensitivity Test used 1000 samples with the inference number of 4 [82]. Derivative-based Global Sensitivity Measure used 1000 samples with finite difference step size of 0.0001 [83]. The result of the sensitivity analysis is shown in Figure 4 and Table A8.

## Appendix C. Variables and Equations

Variables are listed in the order they appear in the Equations.

$t_{b}$ = time of batch (h)
$t_{gf}$ = Time growth phase ends (h)
$t_{m}$ = Time of maturation phase (h)
$F_{c}$ = Final concentration of cells in bioreactor (cells L^−1^)
$B_{v}$ = Bioreactor working volume (L)
$N_{c}$ = Total number of cells in bioreactor (cells)
$V_{c}$ = Volume of single cell (m^3^ cell^−1^)
V = Volume (m^3^)
$\rho_{c}$ = Density of muscle cell (kg m^3^)
$M_{b}$ = mass of ACBM produced per batch (kg batch^−1^)
$b_{BY}$ = Number of batches a single bioreactor can produce in year (batches year^−1^)
$M_{BY}$ = Mass of ACBM a bioreactor can produce in a year (kg year^−1^)
$M_{DY}$ = Desired annual mass of ABCM (kg)
$B_{T}$ = Total number of bioreactors required to annual production goal
$C_{eq}$ = Total equipment costs (USD)
$C_{F}$ = Fixed equipment cost (USD)
$f_{Aj}$ = Adjusted value factor for equipment j
$C_{Uj}$ = Unit costs for equipment j
$U_{j}$ = Base unit for equipment j
$U_{aj}$ = Actual unit for equipment j
$f_{s}$ = Scale factor for equipment j
$f_{L}$ = Lang factor
$f_{FM}$ = Fixed manufacturing cost factor
$C_{FM}$ = Fixed manufacturing costs (USD)
$C_{op}$ = Annual operating costs (USD)
$C_{mY}$ = Total annual costs of media (USD)
$C_{O_{2}Y}$ = Total annual costs of oxygen (USD)
$E_{Hm}$ = Minimum energy required to heat media (kWh)
$E_{BR}$ = Minimum energy required bioreactor heat removal (kWh)
$E_{ACBMR}$ = Minimum annual energy required for ACBM heat removal (kWh)
$C_{L}$ = Estimated annual labor costs (USD)
$C_{E}$ = Cost of energy (cents kWh^−1^)
$C_{W}$ = Annual process water and wastewater costs (USD)
$c_{t}$ = Total number of cells at time (t)
$c_{o}$ = Total number of cells present in inoculum (cells)
$t_{D}$ Doubling time (h)
t = Time (h)
$GCR_{B}$ = Glucose consumption rate within the bioreactor (mol h^−1^)
$GCR_{c}$ = Glucose consumption rate per cell (mol h^−1^ cell^−1^)
$G_{Gg}$ = Total moles of glucose required for growth phase (mol)
$G_{GM}$ = Total moles of glucose required for maturation phase (mol)
$G_{G}$ = Total moles of glucose required per batch (mol)
$m_{ch}$ = Total media charges per batch (charge)
$M_{Gch}$ = Moles of glucose per charge (g)
$V_{b}$ = Total volume of media required per batch (L)
$V_{ch}$ = Volume of charge or bioreactor (L)
$V_{m}$ = Total media volume per year (L year^−1^)
$b_{y}$ = Batches per year
$C_{mL}$ = Cost of media per liter (USD L^−1^)
$OUR_{B}$ = Oxygen uptake rate in bioreactor (mol s^−1^)
$OTR_{B}$ = Oxygen transfer rate in bioreactor (mol s^−1^)
k = mass transfer coefficient (m s^−1^)
A = mean bubble specific interfacial surface area (m^2^)
$e_{con}$ = equilibrium concentration (mol m^−3^)
$a_{con}$ = actual dissolved oxygen concentration (mol m^−3^)
$O_{2}^{i}$ = Initial oxygen in required in the system (mol)
$\rho_{m}$ = Density of media (kg L^−1^)
$P_{O_{2}}$ = Percentage of oxygen (O_2_) in media by weight (%)
$O_{2}^{mol}$ = molar mass of O_2_ (kg mol^−1^)
$OUR_{c}$ = rate of oxygen consumption per cell mol cell^−1^ h^−1^
$O_{2}^{g}$ = Total oxygen required for growth phase per batch (mol)
$O_{2}^{M}$ = Total oxygen required for maturation phase per batch (mol)
$O_{2}^{b}$ = Total oxygen used per ACBM batch (mol)
$O_{2}$ = Total amount of oxygen required per year (mol)
$C_{O_{2Y}}$ = Total annual costs of oxygen (USD)
$C_{O_{2}}$ = Cost of oxygen (USD mol^−1^)
$M_{mY}$ = Mass of media used per year (kg)
ΔT = Temperature difference (°C)
$W_{C_{v}}$ = Specific heat of water at constant volume (kWh kg^−1^ °C^−1^)
$\in_{Hm}$ = Energy efficiency of heating system (%)
$O_{2}$ = Oxygen required annually (mol)
h = Heat released per mol of oxygen consumed (kWh mol^−1^)
$\in_{BR}$ = Energy efficiency of bioreactor cooling system (%)
$ACBM_{C_{v}}$ = Specific heat of ACBM (kWh kg^−1^ °C^−1^)
$\in_{ACBMR}$ = Energy efficiency of ACBM cooling system (%)
$C_{EP}$ = Cost of electricity from a public supplier (USD kWh^−1^)
$C_{NG}$ = Cost of natural gas (USD 1000 ft^−3^)
$C_{bT}$ = Cost of energy from onsite boiler–turbine system (USD kWh^−1^)
$C_{NGP}$ = natural gas price (USD kWh^−1^)
$\epsilon_{bT}$ = boiler–turbine system efficiency (%)
$f_{EP}$ = percentage of electricity produced by from a public supplier (%)
$f_{bT}$ = percentage of energy produced by on site boiler–turbine system (%)
$C_{PW}$ = Process water costs (USD m^−3^)
$C_{WF}$ = Wastewater filtration costs (USD m^−3^)
$C_{BO}$ = Biological oxidation of wastewater costs (USD m^−3^)
P = required manpower (production workers)
$P_{j}$ = production worker required for single piece of equipment
j = Individual piece of equipment
N = All downstream equipment used in downstream ACBM production
$f_{lab}$ = Labor cost correction factor
$f_{C}$ = Country effect
$f_{Sca}$ = Supervising and clerical assistance
$f_{T}$ = Advanced technological and automating
$f_{Q}$ = Skilled and qualified level of the personnel
$f_{B}$ = Social benefits
$f_{O}$ = Overtime work
$C_{Lab}$ = Estimated annual labor costs (USD)
$t_{y}$ = Annual operating time (h)
$C_{L}$ = Production worker hourly rate (USD h^−1^)
$EQ_{r}$ = Equity ratio
$C_{D}$ = Total debt costs (USD)
$D_{r}$ = debt ratio (%)
$C_{TEQ}$ = Total equity costs (USD)
$f_{CRD}$ = Capital recovery factor for debt
$f_{CREQ}$ = Capital recovery factor for equity
$D_{p}$ = Annual debt payment (USD)
$EQ_{p}$ = Annual equity recovery (USD)
$C_{cap}$ = Minimum annual cost of capital expenditures (USD)
$C_{total}$ = Total minimum annual costs (USD)

Equation (A1). Time of batch

(A1) $$t_{b}=t_{gf}+t_{m}$$

Equation (A2). Total number of cells in a single bioreactor after maturation

(A2) $$N_{c}=F_{c}\;B_{V}$$

Equation (A3). Total volume occupied by cells

(A3) $$V=N_{c}\;V_{c}$$

Equation (A4). Cell mass in bioreactor per batch

(A4) $$M_{b}=V\;\rho_{c}$$

Equation (A5). Annual ACBM production per bioreactor

(A5) $$M_{BY}=M_{b}\;b_{BY}$$

Equation (A6). Bioreactors needed to match desired annual beef production

(A6) $$B_{T}=\frac{M_{DY}}{M_{BY}}$$

Equation (A7). Equipment costs equation

(A7) $$C_{eq}=\underset{j}{\sum }f_{Aj}\;C_{Uj}{\left(\frac{U_{aj}}{U_{j}}\right)}^{f_{s}}$$

Equation (A8). Fixed equipment costs

(A8) $$C_{F}=f_{L}\;C_{eq}$$

Equation (A9). Fixed manufacturing costs

(A9) $$C_{FM}=f_{FM\;}C_{F}$$

Equation (A10). Minimum annual operating costs

(A10) $$C_{op}=C_{FM}+C_{mY}+C_{O_{2}Y}+C_{E}\;E_{Hm}+C_{E}\;E_{BR}+C_{E}\;E_{ACBMR}+C_{Lab}+C_{W}$$

Equation (A11). Cells in bioreactor during growth phase

(A11) $$c_{t}=2^{\frac{t}{t_{D}}}\;c_{o}$$

Equation (A12). Glucose consumption rate during growth phase

(A12) $$\frac{dGCR_{B}}{dt}=GCR_{c}\times c_{t}$$

Equation (A13). Total glucose required for growth phase per ACBM batch

(A13) $$G_{Gg}=\int_{t=0}^{t=t_{gf}}GCR_{B}\;dt$$

Equation (A14). Total glucose required for maturation phase per ACBM batch

(A14) $$G_{GM}=GCR_{B}\times t_{m}$$

Equation (A15). Total glucose required per batch

(A15) $$M_{G}=G_{Gg}+G_{GM}$$

Equation (A16). Total required media charges per batch

(A16) $$m_{ch}=G_{G}/G_{Gch}$$

Equation (A17). Total media volume required per batch

(A17) $$V_{b}=m_{ch}\;V_{ch}$$

Equation (A18). Total media volume per year

(A18) $$V_{m}=V_{b}b_{y}$$

Equation (A19). Total annual costs of media

(A19) $$C_{mY}=V_{m}C_{mL}$$

Equation (A20). Oxygen uptake rate

(A20) $$OUR_{B}=OTR_{B}=kA\left(e_{con}-a_{con}\right)$$

Equation (A21). Initial oxygen in the for the system

(A21) $$O_{2}^{i}=\frac{V_{b}\times \rho_{m}\times P_{O_{2}}}{O_{2}^{mol}}$$

Equation (A22). Oxygen uptake rate changing with time

(A22) $$\frac{dOUR_{B}}{dt}=OUR_{c}\times c$$

Equation (A23). Total oxygen required for growth phase per ACBM batch

(A23) $$O_{2}^{g}=\int_{t=0}^{t=t_{gf}}OUR_{B}\;dt$$

Equation (A24). Total oxygen required for maturation phase per ACBM batch

(A24) $$O_{2}^{M}=OUR_{B}\times t_{m}$$

Equation (A25). Total oxygen required per ACBM batch

(A25) $$O_{2}^{b}=O_{2}^{i}+O_{2}^{g}+O_{2}^{M}$$

Equation (A26). Total amount of oxygen required per year

(A26) $$O_{2}=O_{2}^{b}b_{y}$$

Equation (A27). Total annual costs of oxygen

(A27) $$C_{O_{2Y}}=O_{2}C_{O_{2}}$$

Equation (A28). Estimation of energy to heat media to required temperature

(A28) $$E_{Hm}=\frac{M_{mY}\times \Delta T\times W_{C_{v}}}{\in_{Hm}}$$

Equation (A29). Glucose combustion reaction

C_6_H_12_O_6_ + 6 O_2_ → 6CO_2_ + 6 H_2_O + heat
(A29)

Equation (A30). Estimation of energy usage for bioreactor cooling per ACBM batch

(A30) $$E_{BR}=\frac{O_{2}\times h}{\in_{BR}}$$

Equation (A31). Estimation of annual energy usage for cooling of ACBM

(A31) $$E_{ACBMR}=\frac{M_{DY}\times \Delta T\times ACBM_{C_{v}}}{\in_{ACBMR}}$$

Equation (A32). Cost of energy per kWh from public supplier

(A32) $$C_{EP}=0.0969C_{NG}+6.78$$

Equation (A33). Cost of self-generated electric/energy per kWh from a boiler–turbine system

(A33) $$C_{bT}=\frac{C_{NGP}}{\epsilon_{bT}}$$

Equation (A34). Cost of energy per kWh

(A34) $$C_{E}=f_{EP}C_{EP}+f_{bT}C_{bT}$$

Equation (A35). Annual process water and wastewater costs

(A35) $$C_{W}=V_{m}\;C_{PW}+V_{m}\;C_{WF}+V_{m}\;C_{BO}$$

Equation (A36). Required manpower for operation

(A36) $$P=\overset{N}{\underset{j}{\sum }}P_{j}$$

Equation (A37). Labor cost correction factor

(A37) $$f_{lab}=f_{C}f_{Sca}f_{T}f_{Q}f_{B}f_{O}$$

Equation (A38). Estimated annual labor costs

(A38) $$C_{Lab}=t_{y}f_{lab}C_{L}P$$

Equation (A39). Equity ratio

(A39) $$EQ_{r}=100\%-D_{r}$$

Equation (A40). Total debt costs

(A40) $$C_{D}=C_{F}D_{r}$$

Equation (A41). Total equity costs

(A41) $$C_{TEQ}=EQ_{r}\;C_{F}$$

Equation (A42). Capital recovery factor for debt

(A42) $$f_{CRD}=I_{D}{\left(1+I_{D}\right)}^{L_{e}}/\;({\left(1+I_{D}\right)}^{L_{e}-1})$$

Equation (A43). Capital recovery factor for equity

(A43) $$f_{CREQ}=I_{EQ}{\left(1+I_{EQ}\right)}^{L_{e}}/({\left(1+I_{EQ}\right)}^{L_{e}-1})$$

Equation (A44). Annual debt payment

(A44) $$D_{p}=f_{CRD}C_{D}$$

Equation (A45). Annual equity recovery

(A45) $$EQ_{p}=f_{CREq}C_{TEq}$$

Equation (A46). Minimum annual cost of capital expenditures

(A46) $$C_{cap}=D_{p}+Eq_{p}$$

Equation (A47). Total minimum annual cost

(A47) $$C_{total}=C_{cap}+C_{op}$$

## Appendix D. Additional Tables and Figures

Costs comparison of the average United States industrial electricity and natural gas (USD kWh^−1^) 1999–2019. Information was obtained from the United States EIA and average costs were normalized to January 2019 US currency [40,42].

Linear relationship between electricity and natural gas cost. This relationship was used to determine equation 32. Information was obtained from the United States EIA and average costs were normalized to January 2019 US currency [40,42]. Figure produced using Microsoft Excel.

**Table A1:** Model variable inputs: Operations.

**Scenarios**	**Inoculum Concentration (Cells/Ml)**	**Inoculum Bioreactor Volume (L)**	**Seed Bioreactor Volume (L)**	**Seed Bioreactor (Cell/Ml)**	**Bioreactor Volume (M^3^)**	**Desired and Achievable Cell Concentration (Cell/Ml)**	**Desired Mass of Meat Produced (Kg)**
1	1.00 × 10^7^	2.00	2.00 × 10^2^	1.00 × 10^7^	2.00 × 10^1^	1.00 × 10^7^	1.21 × 10^8^
2	9.50 × 10^7^	2.00	2.00 × 10^2^	9.50 × 10^7^	2.00 × 10^1^	9.50 × 10^7^	1.21 × 10^8^
3	9.50 × 10^7^	2.00	2.00 × 10^2^	9.50 × 10^7^	2.00 × 10^1^	9.50 × 10^7^	1.21 × 10^8^
4	2.00 × 10^8^	2.00	2.00 × 10^2^	2.00 × 10^8^	2.00 × 10^1^	2.00 × 10^8^	1.21 × 10^8^
**Scenarios**	**Adjusted Value Factor for Bioreactor**	**Lang Factor**	**Maturation Time (H)**	**Annual Operating Time (H)**	**Bioreactor Scale Factor**	**Fixed Manufacturing Costs Factor**	**Bioreactor Unit Costs (USD/M^3^)**
1	1.29	2.00	240.00	8760.00	0.60	0.15	5.00 × 10^4^
2	1.29	2.00	156.00	8760.00	0.60	0.15	5.00 × 10^4^
3	1.29	2.00	156.00	8760.00	0.60	0.15	5.00 × 10^4^
4	1.29	2.00	24.00	8760.00	0.60	0.15	5.00 × 10^4^

**Table A2:** Model variable inputs: Cell attributes.

Scenarios	Average Single Cell Volume (M^3^/Cell)	Average Single Cell Density (Kg/M^3^)	Hours Per Doubling (H)	Glucose Consumption Rate Per Cell (Mol/H Cell)	Rate of Oxygen Consumption Per Cell (Mol/H Cell)
1	5.00 × 10^−15^	1.06 × 10^3^	24	4.13 × 10^−13^	1.80 × 10^−14^
2	5.00 × 10^−15^	1.06 × 10^3^	16	2.07 × 10^−13^	1.80 × 10^−14^
3	5.00 × 10^−15^	1.06 × 10^3^	16	2.07 × 10^−13^	1.80 × 10^−14^
4	5.00 × 10^−15^	1.06 × 10^3^	8	4.13 × 10^−14^	1.80 × 10^−14^

**Table A3:** Model variable inputs: Media.

**Scenarios**	**Basal Media (USD/L)**	**Ascorbic Acid 2-Phosphate (G/L)**	**Ascorbic Acid 2-Phosphate (USD/G)**	**NAHCO3 (G/L)**		**NAHCO3 (USD/G)**	**Sodium Selenite (G/L)**	**Sodium Selenite (USD/G)**
1	3.12	6.40 × 10^−2^	7.84	5.43 × 10^−1^		0.01	1.40 × 10^−5^	0.10
2	3.12	6.40 × 10^−2^	7.84	5.43 × 10^−1^		0.01	1.40 × 10^−5^	0.10
3	3.12	6.40 × 10^−2^	7.84	5.43 × 10^−1^		0.01	1.40 × 10^−5^	0.10
4	0.24	6.40 × 10^−2^	0.00	5.43 × 10^−1^		0.00	1.40 × 10^−5^	0.00
**Scenarios**	**Insulin (g/L)**	**Insulin (USD/g)**	**Transferrin (g/L)**	**Transferrin (USD/g)**	**FGF-2 (g/L)**	**FGF-2 (USD/g)**	**TGF-b§ (g/L)**	**TGF-b§ (USD/g)**
1	1.94 × 10^2^	340.00	1.07 × 10^2^	400.00	1.00 × 10^−4^	2.01 × 10^6^	2.00 × 10^−6^	8.09 × 10^7^
2	1.94 × 10^2^	340.00	1.07 × 10^2^	400.00	5.00 × 10^−5^	1.00 × 10^6^	2.00 × 10^−6^	8.09 × 10^7^
3	1.94 × 10^2^	340.00	1.07 × 10^2^	400.00	5.00 × 10^−5^	0.00	2.00 × 10^−6^	8.09 × 10^7^
4	1.94 × 10^2^	0.00	1.07 × 10^2^	0.00	0.00	0.00	2.00 × 10^−6^	USD 0.00

**Table A4:** Model variable inputs: Media continued 2.

Scenarios	Percentage of Oxygen in Initial Charge (*W*/*W*)	Oxygen (USD/Ton)	Glucose (Mol/L)	Density of Media (Kg/L)
1	2.00	4.00 × 10^1^	1.78 × 10^−2^	1.00
2	2.00	4.00 × 10^1^	2.67 × 10^−2^	1.00
3	2.00	4.00 × 10^1^	2.67 × 10^−2^	1.00
4	2.00	4.00 × 10^1^	3.56 × 10^−2^	1.00

**Table A5:** Model variable inputs: Utility.

**Scenarios**	**Boiler Energy Efficiency (%)**	**Percentage of Electricity Self-Generated (%)**	**Temperature of Water/Media Entering Facility (°C)**	**Desired Temperature of Media Entering Bioreactor (°C)**		**Specific Heat of Water (Kwh/Kg (°C))**	**Energy Efficiency of Media Heating System (%)**	**Heat Released Per Mol of Oxygen Consumed (Kwh)**	**Energy Efficiency of Bioreactor Cooling System (%)**
1	85	50	20	37		1.16 × 10^−3^	100	1.30 × 10^−1^	100
2	85	50	20	37		1.16 × 10^−3^	100	1.30 × 10^−1^	100
3	85	50	20	37		1.16 × 10^−3^	100	1.30 × 10^−1^	100
4	85	50	20	37		1.16 × 10^−3^	100	1.30 × 10^−1^	100
**Scenarios**	**Specific Heat of ACBM (Kwh/Kg °C)**	**Temperature of ACBM In Bioreactor (°C)**	**Temperature of Cooled ACBM (°C)**	**Energy Efficiency of ACBM Cooling System (%)**	**Natural Gas Cost (U.S. Dollars Per 1000 Ft^3^)**	**Natural Gas (U.S. Dollars Per Kwh)**	**Process Water Cost (USD/M^3^)**	**Wastewater Filtration Treatment Costs (USD/M^3^)**	**Biological Oxidation of Wastewater Costs (USD/M^3^)**
1	6.22 × 10^−4^	37	4	100	4.17	0.0142	0.63	0.51	0.57
2	6.22 × 10^−4^	37	4	100	4.17	0.0142	0.63	0.51	0.57
3	6.22 × 10^−4^	37	4	100	4.17	0.0142	0.63	0.51	0.57
4	6.22 × 10^−4^	37	4	100	4.17	0.0142	0.63	0.51	0.57

**Table A6:** Model variable inputs: Labor.

Scenarios	Production Worker Hourly Rate (USD/H)	Country Effect	Supervising and Clerical Assistance	Advanced Technology and Automating	Skilled and Qualified Level of The Personnel	Social Benefits	Overtime Work	Bioreactors Labor Factor
1	13.68	1.00	1.20	0.80	1.50	1.40	1.25	1.00
2	13.68	1.00	1.20	0.80	1.50	1.40	1.25	1.00
3	13.68	1.00	1.20	0.80	1.50	1.40	1.25	1.00
4	13.68	1.00	1.20	0.80	1.50	1.40	1.25	1.00

**Table A7:** Model variable inputs: Finance.

Scenarios	Debt Ratio (%)	Interest Rate on Debt (%/Y)	Economic Life (Y)	Interest Cost of Equity (%/Y)
1	90	5	20.00	15
2	90	5	20.00	15
3	90	5	20.00	15
4	90	5	20.00	15

Model variable inputs. Inputs without unit in parentheses are unitless.

**Table A8:** Sensitivity analysis numerical results.

Algorithm	Average Single Cell Density (rho_c)	Average Single Cell Volume (V_c)	Glucose Conc. (conc_glu)	Glucose Consumption Rate Per Cell (GCR_c)	FGF-2 Cost (C_fgf2)	FGF-2 Conc. (conc_fgf2)	Maturation Time (t_m)	TGF-β Conc. (conc_tgfb)	Oxygen Consumption Rate Per Cell (OUR_c)
DGSM	6.83 × 10^3^	1.00 × 10^0^	2.70 × 10^−2^	5.70 × 10^−1^	2.40 × 10^−3^	5.07 × 10^−2^	8.03 × 10^−3^	4.93 × 10^−2^	8.68 × 10^−2^
SSA	1.00 × 10^0^	9.66 × 10^−1^	9.48 × 10^−1^	8.80 × 10^−1^	8.50 × 10^−1^	7.47 × 10^−1^	6.95 × 10^−1^	2.16 × 10^−3^	1.69 × 10^−3^
DMIM	8.90 × 10^−1^	1.00 × 10^0^	9.47 × 10^−1^	7.58 × 10^−1^	7.83 × 10^−1^	9.10 × 10^−1^	5.98 × 10^−1^	1.37 × 10^−2^	5.13 × 10^−2^
FAST	7.82 × 10^−1^	1.00 × 10^0^	5.83 × 10^−1^	8.63 × 10^−1^	4.97 × 10^−1^	8.50 × 10^−1^	6.94 × 10^−1^	1.59 × 10^−4^	1.93 × 10^−6^
MM	1.00 × 10^0^	9.70 × 10^−1^	9.91 × 10^−1^	9.53 × 10^−1^	9.11 × 10^−1^	9.09 × 10^−1^	8.62 × 10^−1^	1.44 × 10^−2^	1.44 × 10^−8^
RBD-FAST	1.00 × 10^0^	7.94 × 10^−1^	9.96 × 10^−1^	7.54 × 10^−1^	7.86 × 10^−1^	7.11 × 10^−1^	8.22 × 10^−1^	1.39 × 10^−1^	7.48 × 10^−2^

Sensitivity analysis numerical results. DGSM = Derivative-based Global Sensitivity Measure, SSA = Sobol Sensitivity Analysis, DMIM = Delta Moment-Independent Measure, FAST = Fourier Amplitude Sensitivity Analysis MM = Morris Method and RBD-FAST = Random Balance Designs-Fourier Amplitude Sensitivity Test. The 5 parameters exhibiting the most sensitivity were selected from each algorithm. This resulted in 9 unique parameters listed in the table. This analysis was performed using peer reviewed open-source SALib Python package for this work [49].

**Table A9:** Potential industrial scale equipment for ACBM production.

Equipment	Unit	Unit Costs (USD 1000’s)	Scale Index	Production Operators Required (P)	Adjusted Value Factor (F_aj_)	Accounted for in Equipment Cost Analysis
Centrifugal pumps	Power (kW)	5	0.60	0.1	1.42	-
Plate filters	Area (m^2^)	3	0.75	1.0	1.64	-
Media holding vessel	Volume (m^3^)	10	0.50	0.2	1.29	-
Heat exchanger	Area (m^2^)	3	0.65	0.5	1.29	-
Inoculum bioreactor	Volume (m^3^)	50	0.60	1.0	1.29	-
Seed bioreactor	Volume (m^3^)	50	0.60	1.0	1.29	-
Bioreactors	Volume (m^3^)	50	0.60	1.0	1.29	+
Positive displacement pump	Power (kW)	5	0.60	0.1	1.42	-

Potential industrial scale equipment for ACBM production. Created using information from *Food Plant Economics* and CEPI [31,32,44].

**Table A10:** Annual United States national industrial grid electricity costs 1999–2019.

Year	Average Nominal Consumer Cost Per Year (Cents Kwh^−1^)	Inflation Adjusted Cost (Cents Kwh^−1^)
1999	4.42	6.77
2000	4.63	6.9
2001	5.04	7.25
2002	4.88	6.94
2003	5.11	7.08
2004	5.25	7.14
2005	5.72	7.59
2006	6.15	7.81
2007	6.39	7.95
2008	6.95	8.29
2009	6.83	8.14
2010	6.76	7.85
2011	6.81	7.78
2012	6.66	7.4
2013	6.88	7.52
2014	7.09	7.63
2015	6.90	7.43
2016	6.75	7.17
2017	6.87	7.12
2018	6.92	7.03

Annual United States industrial national grid electricity costs 1999–2019. Information was obtained from the United States EIA and average costs were normalized to January 2019 US currency [40,42].

**Table A11:** Annual United States national industrial natural gas costs 1999-2019.

Year	Average Nominal Cost Per Year (USD Thousand Cubic Feet^−1^)	Inflation Adjusted Cost (Cents Kwh^−1^)
1999	3.08	1.55
2000	4.45	2.19
2001	5.08	2.40
2002	4.02	1.88
2003	5.91	2.70
2004	6.51	2.92
2005	8.67	3.77
2006	7.82	2.58
2007	7.65	3.13
2008	9.66	3.79
2009	5.23	2.05
2010	5.44	2.08
2011	5.12	1.93
2012	3.85	1.41
2013	4.64	1.67
2014	5.58	1.98
2015	3.91	1.39
2016	3.49	1.22
2017	4.08	1.39
2018	4.17	1.42

Annual United States national average natural gas costs 1999–2019. Information was obtained from the United States EIA and average costs were normalized to January 2019 US currency [40,42].

**Table A12:** Cost of process and wastewater treatment.

Utility	Cost (USD m^−3^)
Process water	0.63
Wastewater filtration treatment	0.51
Biological oxidation of wastewater	0.57

Cost of process and wastewater treatment. Cost were reported in *Food Plant Economics* and were adjusted to account for inflation reported in January 2019 US currency [42,44].
